# 1700. Posaconazole-Induced Excess Mineralocorticoid Syndrome with Hypertension, Hypokalemia, and Inhibition of 11-b-hydroxylase in Pediatric Patients

**DOI:** 10.1093/ofid/ofad500.1533

**Published:** 2023-11-27

**Authors:** Tempe K Chen, Jagmohan S Batra, Rachit Chawla, Natalie Quanquin, David E Michalik, Kavita Sharma, Cristina Farkas-Skiles, Bhavita Patel, Jacqueline Casillas, Ramesh Patel, Jong Chung, Meena Kadapakkam, Maki Okada, Thomas J Walsh

**Affiliations:** Miller Children's and Women's Hospital Long Beach, Long Beach, California; University of California, Irvine School of Medicine, Long Beach, California; St. John's Community Health Inc., Los Angeles, California; Miller Children's and Women's Hospital Long Beach, Long Beach, California; University of California, Irvine School of Medicine, Long Beach, California; Miller Children's and Women's Hospital Long Beach, Long Beach, California; Miller Children's and Women's Hospital Long Beach, Long Beach, California; University of California, Los Angeles David Geffen School of Medicine, Long Beach, California; University of California, Los Angeles David Geffen School of Medicine, Long Beach, California; Miller Children's Hospital Long Beach, Long Beach, California; University of California, Los Angeles David Geffen School of Medicine, Long Beach, California; University of California, Los Angeles David Geffen School of Medicine, Long Beach, California; Miller Children's and Women's Hospital Long Beach, Long Beach, California; Center for Innovative Therapeutics and Diagnostics and University of Maryland School of Medicine, Richmond, Virginia

## Abstract

**Background:**

Posaconazole is a potent broad spectrum mould-active triazole that is increasingly used in children for treatment of aspergillosis, mucormycosis, and endemic mycoses. Although posaconazole has a favorable safety profile in pediatric patients, we recently observed an excess mineralocorticoid syndrome characterized by hypertension and hypokalemia within three weeks of treatment initiation in two patients. Both showed endocrinological evidence of posaconazole inhibition of 11-β-hydroxylase. As this condition is seldom reported in children, we conducted a systematic review of the literature for reports of this condition in pediatric cases.

**Methods:**

A systematic review of the literature (https://pubmed.ncbi.nlm.nih.gov/ and https://scholar.google.com/) was performed using key phrases of pediatrics (< 18 years) plus posaconazole plus hypertension, hypokalemia, mineralocorticoid excess, or 11-β-hydroxylase. Variables included age, sex, underlying condition, indication for posaconazole, blood pressure > 95th% for age and height, time from exposure to posaconazole to onset of hypertension, hypokalemia (≤3mEq/L), plasma aldosterone, serum 11- deoxycorticosterone, and serum 11-deoxycortisol.

**Results:**

The systematic literature review identified three reported cases. Clinical characteristics and laboratory data from all five cases are summarized in the table. Median age was 7 yrs (range: 5-13 yrs). Four of 5 patients were male. Patients were treated for mucormycosis, histoplasmosis or ABPA. All patients developed hypertension within three weeks of starting posaconazole. Hypokalemia occurred in 4 out of 5 cases. Consistent with posaconazole inhibition of 11-β-hydroxylase, patients with available data had elevated serum 11- deoxycorticosterone, and/or elevated serum 11-deoxycortisol, and/or decreased or undetectable plasma aldosterone. All patients were managed with antihypertensive therapy.

**Figure:**
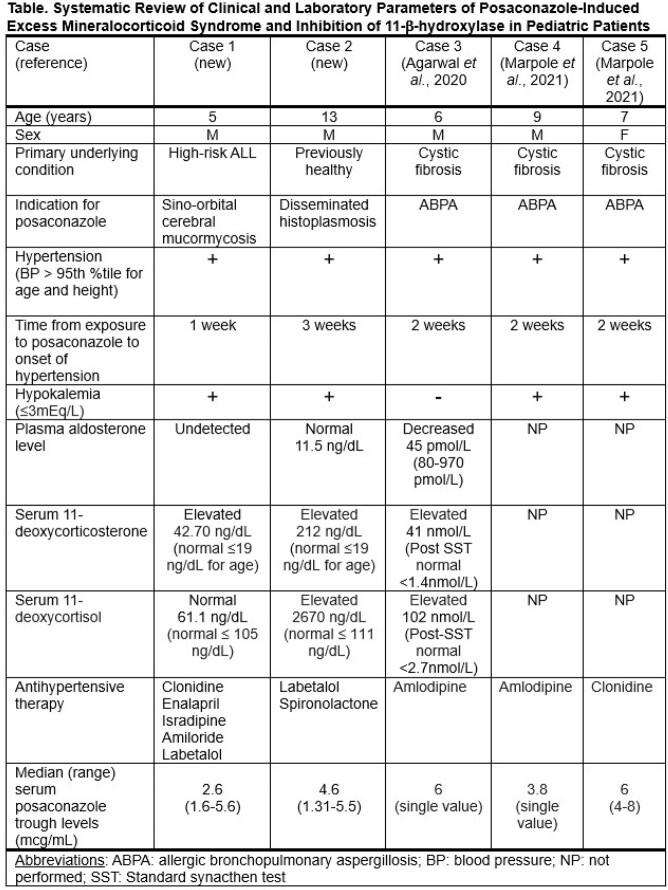

**Conclusion:**

Children who develop hypertension and hypokalemia within 3 weeks of receiving posaconazole should be evaluated further for an excess mineralocorticoid syndrome and 11-β-hydroxylase inhibition. Management may consist of discontinuation of posaconazole and/or initiation of antihypertensive therapy.

**Disclosures:**

**Jong Chung, MD**, Cardinal Health: Advisor/Consultant|Dispersol Technologies: Advisor/Consultant|Forma Therapeutics: Advisor/Consultant|Global Blood Therapeutics: Advisor/Consultant|Jazz Pharmaceuticals: Advisor/Consultant **Thomas J. Walsh, MD PhD**, Abbott: Advisor/Consultant|Amplyx: Grant/Research Support|Astellas: Advisor/Consultant|Astellas: Grant/Research Support|F2G: Advisor/Consultant|F2G: Grant/Research Support|Gilead: Advisor/Consultant|Gilead: Grant/Research Support|Karyopharm: Advisor/Consultant|Lediant: Advisor/Consultant|Lediant: Grant/Research Support|Merck: Grant/Research Support|Omeros: Advisor/Consultant|Omeros: Grant/Research Support|Partner Therapeutics: Advisor/Consultant|Scynexis: Advisor/Consultant|Scynexis: Grant/Research Support|Shionogi: Advisor/Consultant|Shionogi: Grant/Research Support|Statera: Advisor/Consultant|T2 Biosystems: Advisor/Consultant|T2 Biosystems: Grant/Research Support

